# Risk of autoimmune rheumatic diseases in patients with palindromic rheumatism: A nationwide, population-based, cohort study

**DOI:** 10.1371/journal.pone.0201340

**Published:** 2018-07-26

**Authors:** Hsin-Hua Chen, Wen-Cheng Chao, Tsai-Ling Liao, Ching-Heng Lin, Der-Yuan Chen

**Affiliations:** 1 Department of Medical Research, Taichung Veterans General Hospital, Taichung, Taiwan; 2 Division of Allergy, Immunology, and Rheumatology, Department of Internal Medicine, Taichung Veterans General Hospital, Taichung, Taiwan; 3 School of Medicine, National Yang-Ming University, Taipei, Taiwan; 4 Institute of Biomedical Science and Rong-Hsing Research Center for Translational Medicine, Chung-Hsing University, Taichung, Taiwan; 5 School of Medicine, Chung-Shan Medical University, Taichung, Taiwan; 6 Institute of Public Health and Community Medicine Research Center, National Yang-Ming University, Taipei, Taiwan; 7 Division of Chest Medicine, Department of Internal Medicine, Taichung Veterans General Hospital, Taichung, Taiwan; 8 Department of Medical Education, Taichung Veterans General Hospital, Taichung, Taiwan; Soroka University Medical Center, ISRAEL

## Abstract

**Objective:**

To estimate the relative risk of autoimmune rheumatic diseases, including rheumatoid arthritis (RA), systemic lupus erythematosus (SLE), systemic sclerosis (SSc), Sjogren’s syndrome (SS), dermatomyositis (DM) and polymyositis (PM), among patients with palindromic rheumatism (PR) compared with non-PR individuals.

**Methods:**

The study utilized 2003–2013 claims data from the Taiwanese National Health Insurance Research Database. We identified 4,421 cases of PR from 2007 to 2012 and randomly chose 44,210 non-PR individuals who matched (1:10) for age, sex and the year of index date without prior history of RA, SLE, SSc, SS, DM, or PM. After adjusting for age, sex, and the Charlson comorbidity index, we calculated the hazard ratios (HRs) with 95% confidence intervals (CIs) using the Cox proportional hazard model to quantify the risk of RA, SLE, SS, DM and PM in PR patients compared with that in matched non-PR individuals.

**Results:**

Among the 4,421 patients with PR, 569 (12.87%) developed RA, 269 (6.08%) developed SS, 113 (2.56%) developed SLE, 5 (0.11%) developed SSc, 8 (0.18%) developed PM, and 1 (0.02%) developed DM. After adjusting for potential confounders, the patients with PR had an increased risk of RA (HR, 118.76; 95% CI, 89.81–157.04), SS (HR, 59.57; 95% CI, 43.87–80.88), SLE (HR, 51.56; 95% CI, 32.96–80.66) PM (HR, 57.38; 95% CI, 6.90–476.83), and SSc (HR, 13.42; 95% CI, 3.79–47.55) but not of DM (HR, 3.44; 95% CI, 0.34–34.59).

**Conclusion:**

Patients with PR had an increased risk of developing RA, SS, SLE, PM, and SSc.

## Introduction

Palindromic rheumatism (PR) is a clinical disorder initially described in 1941 by Hernch and Rosenberg [1]. It is characterized by the episodic acute onset of para-arthritis or arthritis, involving one or several joints with variable and irregular symptom-free intervals [1, 2]. Each attack of PR usually lasts from a few hours to days, but rarely over one week [2]. Any joint may be affected, but the hands, wrists, shoulders and knees are more frequently involved [2, 3]. An attack of PR is usually afebrile and subsides spontaneously. The diagnosis of PR is based on the exclusion of other types of arthritis. The diagnostic criteria for PR were proposed by Guerne and Weismann in 1992 [4], consisting of the above characteristics with at least a six-month history and evidence of an attack observed by a physician.

The exact etiopathogenesis of PR remains unclear. PR has been found to be associated with the development of autoimmune rheumatic diseases [5], particularly rheumatoid arthritis (RA) [6]. After a variable period of follow-up, approximately 15%–66% of patients with PR were reported to have developed RA [5, 7–12]. Predictors of the progression of PR to RA include the genetic background [13, 14], ultrasonographic findings of synovitis, presence of anti-cyclic citrullinated peptide antibodies (ACPA) [8, 15], positive rheumatoid factor [5], and hand involvement [5, 16]. On the contrary, some genetic background, such as HLA-DRB1 *0803 [17], show an increased susceptibility to PR but not to RA, suggesting that PR is distinct from RA.

Although the association between PR and RA development has been widely reported, no research has estimated the magnitude of the risk of progression of PR to RA or to other autoimmune rheumatic diseases, such as systemic lupus erythematosus (SLE), systemic sclerosis (SSc), Sjogren’s syndrome (SS), dermatomyositis (DM) and polymyositis (PM). This study aimed to examine the relative risk of development of RA, SLE, SSc, SS, DM, or PM among patients with PR compared with that in non-PR individuals using a nationwide, population-based, administrative dataset.

## Methods

### Ethics statement

The Institutional Review Board (IRB) of the Taichung Veterans General Hospital (IRB number: CE17156A) approved this study. We did not acquire an informed consent due to the anonymization of personal information before the analysis of the data.

### Study design

The study used a retrospective cohort design.

### Data source

The data were extracted from the 2003–2013 Taiwanese National Health Insurance Research Database (NHIRD). In Taiwan, a compulsory National Health Insurance (NHI) program was initiated in 1995, which presently covers over 99% of the Taiwanese population. NHIRD comprises claims data regarding inpatient, outpatient, and traditional medicine services and detailed drug prescription information. However, data regarding some personal history, such as body mass index (BMI), tobacco and alcohol use, are not included in NHIRD. The accuracy of NHIRD has been improved by regular checks of original medical records by the Bureau of NHI (BNHI) [18]. Researchers are allowed to apply the database for study purpose from the National Health Research Institute (NHRI), which processes NHIRD data and anonymizes personal information.

This study utilized the 2003–2013 administrative data regarding outpatient services, inpatient services, and enrollment files from NHIRD to select all patients with newly diagnosed PR during 2007–2012 as the study cohort. NHRI randomly selected one million enrollees from the entire Taiwanese population who received NHI in 2000 and constructed a representative longitudinal health insurance database (LHID2000) of these enrollees, from which the comparison cohort was selected. The data of the comparison cohort that were used for analysis were extracted from LHID2000.

BNHI has established a registry for catastrophic illness patients (RCIP), who have major or severe illnesses, including cancer and certain connective tissue diseases, such as RA, SLE, SSc, SS, DM, and PM. It has issued a certificate for “catastrophic illness” for patients diagnosed with major or severe diseases, which were validated by at least two qualified corresponding specialists after a careful review of their original medical charts. The patients who possessed a certificate for “catastrophic illness” were exempted from copayment for ambulatory or inpatient visits.

### Definition of PR

Patients were identified as PR cases if they had ≥3 ambulatory visits or ≥1 hospital admission with a PR diagnosis (International Classification of Diseases, Ninth Revision, Clinical Modification [ICD-9-CM] code 719.3) during 2003–2013. The diagnosis of PR in Taiwan was based on the classification criteria proposed by Guerne and Weismann in 1992 [4].

### Outcome

The outcome of this study was the time from the index date to the development of various major autoimmune diseases (RA, SLE, SSc, SS, DM, or PM). Patients who developed major autoimmune diseases were defined as being registered in RCIP for the diagnosis of RA (ICD-9-CM code 714.0), SLE (ICD-9-CM code 710.0), SSc (ICD-9-CM code 710.1), SS (ICD-9-CM code 710.2), DM (ICD-9-CM code 710.3), or PM (ICD-9-CM code 710.4) after the index date. The date of enrollment in RCIP for the corresponding major autoimmune disease was defined as the index date of the major autoimmune disease diagnosis.

### Study subjects

#### Newly diagnosed PR patients identified from the entire Taiwanese population

All newly diagnosed PR patients from 2007 to 2012 were included in the study. Patients who were first diagnosed with PR before January 1, 2007 were excluded. The index date of PR cases was defined as the date of the first outpatient or inpatient visit with a diagnosis of PR. We also excluded patients who were registered in RCIP with a diagnosis of RA, SLE, SS, SSc, PM or DM before the index date.

#### Matched non-PR comparison group randomly selected from the representative population of one million enrollees

The non-PR individuals were defined as having no ambulatory or inpatient diagnosis of PR during 2003–2013. We randomly selected non-PR individuals from LHID2000. We used propensity score matching (1:10) to include the baseline differences between individuals with and without PR. The propensity score was estimated using a multivariable logistic regression model, which included sex, age, and the year of the index date (index year). We used the date of the first outpatient or inpatient visit in the index year due to any reason as the index date for the non-PR group. Patients registered in the RCIP for RA, SLE, SS, SSc, PM, or DM before the index date were excluded from the non-PR comparison group.

### Confounders

The association between PR and autoimmune rheumatic diseases was adjusted for potential confounders, including age, sex, and the Charlson comorbidity index (CCI) (0, ≥1). We used CCI adapted by Deyo et al. [19] to represent the overall level of common comorbid illnesses. We identified patients with comorbidity used to calculated CCI if they had three or more ambulatory visits or at least one hospitalization with the corresponding ICD-9-CM code within one year before the index date.

### Subgroup analyses

We performed subgroup analyses based on age (<65 years, ≥65 years), sex, and CCI (0, ≥1) to examine the consistency of results across the subgroups as well as the modification effect of age, sex, or CCI on the association between PR and the development of autoimmune rheumatic diseases.

### Statistical analysis

We presented continuous variables as mean ± standard deviation and categorical variables as percentage of patients. We examined the differences for continuous variables using the Student’s *t*-test and for categorical variables using Pearson’s χ^2^ test. We quantified the associations between PR and the risk of developing autoimmune rheumatic diseases by calculating hazard ratios (HRs) with 95% confidence intervals (CIs) using the Cox proportional regression analysis after adjusting for the confounders. We tested the significance of the interaction effect of age, sex, and CCI on the association between PR and the risk of autoimmune rheumatic diseases by the estimating the *p*-value of the coefficient associated with the product of the indicator of age, sex, or CCI and the indicator of PR using the Wald test. We considered a two-tailed *p*-value of <0.05 as statistically significant. We performed all statistical analyses using the SAS statistical software, version 9.3 (SAS Institute, Inc., Cary, NC, USA).

## Results

A total of 4,421 incident PR patients were included as the PR cohort, and 44,210 non-PR individuals were selected as the comparison cohort matching (1:10) for age, sex, and the year of the initial PR diagnosis date (index date). As shown in Table 1, the mean age ± SD was 46 ± 15 years, and 70.4% of the study subjects were female. The proportion of patients with one or more CCI was higher in the PR group than in the non-PR group.

**Table 1:** Demographic data and clinical characteristics among patients.

	Non-PR	PR	
	(*n* = 44,210)	(*n* = 4,421)	*p*-value
**Age, years** (mean ± SD)	46 ± 15	46 ± 15	1.000
<65 years	39,120 (88.5)	3,912 (88.5)	
≥65 years	5,090 (11.5)	509 (11.5)	
**Sex**			1.000
Female	31,130 (70.4)	3,113 (70.4)	
Male	13,080 (29.6)	1,308 (29.6)	
**CCI** (mean ± SD)	0.25 ± 0.78	0.48 ± 0.90	<0.001
**CCI group**			<0.001
0	37,658 (85.2)	2,963 (67.0)	
≥1	6,552 (14.8)	1,458 (33.0)	

The results are shown as a number (%) unless specified otherwise.

Abbreviations: PR, palindromic rheumatism; SD, standard deviation; CCI, Charlson comorbidity index.

Table 2 compared the incidence rates of the development of various autoimmune rheumatic diseases between the PR patients and non-PR individuals. In the PR patients, the incidence of a major autoimmune disease was the highest for RA (4,433 per10^5^ years), followed by SS (1,985 per10^5^ years), and SLE (812 per10^5^ years). Compared with non-PR individuals, the PR patients had significantly higher incidence rates of RA, SLE, SSc, SS, and PM but not DM.

**Table 2:** Comparison of the incidence rates of developing various autoimmune diseases between the PR patients and non-PR individuals.

Autoimmune disease	Total	Event (%)	Total person-years	Incidence Rate (/10^5^ years)	IRR (95% CI)
**RA**					
Non-PR	44,210	55 (0.12)	156,433	35	Reference
PR	4,421	569 (12.87)	12,806	4,443	126.38 (95.83–166.68)
**SLE**					
Non-PR	44,210	24 (0.05)	156,544	15	Reference
PR	4,421	113 (2.56)	13,909	812	52.99 (34.11–82.33)
**SSc**					
Non-PR	44,210	5 (0.01)	156,573	3	Reference
PR	4,421	5 (0.11)	14,198	35	11.03 (3.19–38.09)
**SS**					
Non-PR	44,210	50 (0.11)	156,455	32	Reference
PR	4,421	269 (6.08)	13,555	1,985	62.10 (45.92–83.98)
**DM**					
Non-PR	44,210	3 (0.01)	156,576	2	Reference
PR	4,421	1 (0.02)	14,213	7	3.67 (0.38–35.30)
**PM**					
Non-PR	44,210	1 (0.00)	156,578	1	Reference
PR	4,421	8 (0.18)	14,203	56	88.20 (11.03–705.18)

The matched variables include age, sex and year of the index date. The adjusted variable includes age, sex, and Charlson comorbidity index (0, ≥1).

Abbreviations: PR, palindromic rheumatism; RA, rheumatoid arthritis; SLE, systemic lupus erythematosus; SSc, systemic sclerosis; SS, Sjogren’s syndrome; DM, dermatomyositis; PM, polymyositis; CI, confidence interval.

As shown in Table 3, the PR patients had a significantly higher risk of developing RA, SLE, SSc, SS or PM, but not DM, than the non-PR individuals. The results were consistent after adjusting for age, sex, and CCI.

**Table 3:** Univariate and multivariable analyses for the relative risk of the development of various autoimmune rheumatic diseases in patients with PR compared with non-PR individuals.

Autoimmune disease	Univariate analysis			Multivariable analysis		
	HR	(95% CI)	*p*-value	HR	(95% CI)	*p*-value
**RA**						
Non-PR	Reference			Reference		
PR	118.18	(89.54–155.97)	<0.001	118.76	(89.81–157.04)	<0.001
**SLE**						
Non-PR	Reference			Reference		
PR	51.46	(33.10–80.00)	<0.001	51.56	(32.96–80.66)	<0.001
**SSc**						
Non-PR	Reference			Reference		
PR	11.44	(3.27–40.00)	<0.001	13.42	(3.79–47.55)	<0.001
**SS**						
Non-PR	Reference			Reference		
PR	59.08	(43.66–79.94)	<0.001	59.57	(43.87–80.88)	<0.001
**DM**						
Non-PR	Reference			Reference		
PR	3.50	(0.36–33.64)	0.278	3.44	(0.34–34.59)	0.295
**PM**						
Non-PR	Reference			Reference		
PR	84.49	(10.57–675.61)	<0.001	57.38	(6.90–476.83)	<0.001

Abbreviations: PR, palindromic rheumatism; HR, hazard ratio; CI, confidence interval; PR, RA, rheumatoid arthritis; SLE, systemic lupus erythematosus; SSc, systemic sclerosis; SS, Sjogren’s syndrome; DM, dermatomyositis; PM, polymyositis.

Multivariable analysis: adjusted for the patient’s age, sex, and Charlson comorbidity index (0, ≥1).

**Fig 1:**
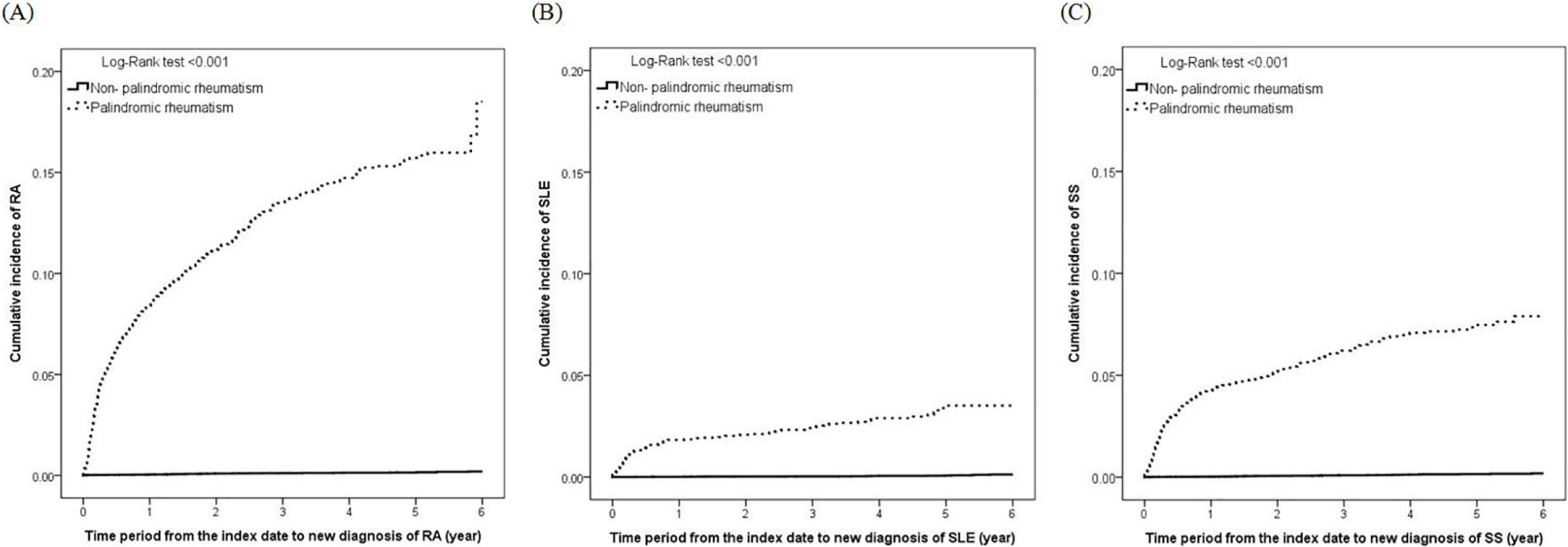
**The cumulative incidences of RA (A), SLE (B), and SS (C) in the PR group and the non-PR group**.

As shown in Fig 1, the cumulative incidences of RA, SLE, and SS were all significantly higher in the PR group than in the non-PR group (*p* <0.001 by the Log-Rank test).

As shown in Table 4, the associations between PR and the risk of developing RA, SLE, or SS remained statistically significant among the subgroups stratified by age, sex, or CCI. Also, the association between PR and the risk of RA was significantly stronger among those with CCI = 0 than among those with CCI ≥1 (*p* for interaction <0.001). The associations between PR and the risk of SLE were significantly stronger among those aged <65 years than among those aged ≥65 years (*p* for interaction = 0.022), and also stronger among those with CCI = 0 than among those CCI ≥1 (*p* for interaction = 0.007). The associations between PR and the risk of SS were also significantly stronger among those aged <65 years than among those aged ≥65 years (*p* for interaction = 0.008), and also stronger among those with CCI = 0 than among those CCI ≥1 (p for interaction <0.001).

**Table 4:** Multivariable analyses for associations between PR and the risk of RA, SLE, or SS among subgroups according to age, sex, and CCI.

Subgroup	RA		SLE		SS	
	HR (95% CI)	*p*-value*	HR (95% CI)	*p*-value*	HR (95% CI)	*p*-value*
**Age**		0.187		0.022		0.008
<65 years	124.78 (92.59–168.18)		61.83 (38.16–100.17)		70.01 (49.72–98.56)	
≥65 years	69.66 (31.23–155.38)		10.11 (2.48–41.20)		22.05 (10.72–45.33)	
**Sex**		0.886		0.412		0.315
Female	117.65 (85.47–161.94)		50.44 (31.88–79.79)		56.24 (40.95–77.22)	
Male	119.01 (66.88–211.78)		111.60 (14.21–876.76)		103.83 (31.41–343.24)	
**CCI**		<0.001		0.007		<0.001
0	171.22 (120.47–243.34)		69.87 (41.46–117.76)		86.55 (59.15–126.63)	
≥1	38.52 (24.44–60.72)		18.12 (7.87–41.72)		20.01 (12.07–33.16)	

*p for interaction

Abbreviations: PR, palindromic rheumatism; RA, rheumatoid arthritis; SLE, systemic lupus erythematosus; SS, Sjogren’s syndrome; HR, hazard ratio; CCI, Charlson comorbidity index.

As shown in Table 5, the association between PR and the risk of developing SSc remained statistically significant among those aged <65 years, the female subjects, or those with CCI = 0. The association between PR and the risk of PM remained statistically significant among only those aged <65 years, the female subjects, or those with CCI ≥1. However, PR was not significantly associated with the development of DM in all of the subgroups.

**Table 5:** Multivariable analyses for associations between palindromic rheumatism and the risk of SSc or PM among subgroups according to age, sex, and CCI.

	SSc		PM	
	HR (95%CI)	*p*-value*	HR (95%CI)	*p*-value*
**Age group**		0.992		1.000
<65 years	16.42 (4.41–61.17)		53.70 (6.45–447.40)	
≥65 years	<0.01 (0.00–∞)		^#^	
**Sex**		0.989		0.994
Female	10.47 (2.72–40.27)		41.36 (4.86–351.87)	
Male	4.5*10^11^ (0.00–∞)		4.7*10^11^ (0.00–∞)	
**CCI**		0.989		0.991
0	16.42 (4.41–61.17)		6.5*10^11^ (0.00–∞)	
≥1	<0.01 (0.00–∞)		19.45 (2.27–166.49)	

*p for interaction

^#^None of the subjects aged ≥65 years in the PR group or non-PR group developed polymyositis.

Abbreviations: SSc, systemic sclerosis; PM, polymyositis; CCI, Charlson comorbidity index; HR, hazard ratio.

## Discussion

This study aimed to estimate the relative risk of developing autoimmune rheumatic diseases, including RA, SLE, SS, SSc, PM, and DM, among PR patients compared with non-PR individuals. We found statistically significant associations between PR and the risk of RA, SLE, SS, SSc, and PM, but not DM, after an adjustment for age, sex, and CCI. Although the proportion of the development of autoimmune diseases was the highest for RA (12.87%), followed by SS (6.08%), SLE (2.56%), PM (0.18%), SSc (0.11%) and DM (0.02%), the magnitude of the relative risk was greatest for RA (HR, 118.76), followed by SS (HR, 59.57), PM (HR, 57.38), SLE (HR, 51.56), and SSc (HR, 13.42). Although the association between PR and the risk of RA had been demonstrated, this study calculated the relative risk of RA development in PR patients compared with non-PR individuals. Previous studies showed that one to two-thirds of the PR patients progressed to RA during the period of their follow-up [2, 7–11]. However, our present study showed that only 12.9% of the PR patients developed RA after a mean duration of 1.0 year. The presence of rheumatoid factor (RF) and ACPA have been revealed as predictors for the progression to RA in PR patients [5, 9, 11, 12, 15, 20]. Although RF and ACPA were not available in the NHIRD, our previous hospital-based study showed a low positive rate of ACPA (13%) and RF (14%) among Taiwanese PR patients, lower than those reported in other regions [5, 9, 11, 12, 20]. Given a similar rate of progression to RA in the PR patients in the present study compared with that in our prior hospital-based one (15.0% after a mean duration of 1.4 years) [15], the low rate of progression to RA in the PR patients might be explained by a low proportion of patients with a positive RF or ACPA.

This study also demonstrated a strong association between PR and the risk of autoimmune rheumatic diseases other than RA. A possible explanation for such an association is a shared genetic background. Kim et al. reported that patients with HLA-DRB1 0803 were susceptible to PR development, and HLA-DRB1 1302 was associated with a decreased risk of PR [17]. Previous studies also showed an association between HLA-DRB1 0803 with the development of SS [21], SLE [22], SSc [23], PM [24], and idiopathic inflammatory myopathy (i.e., PM, DM, or myositis overlapping with other collagen vascular disease) [25]. The non-significance of the association between PR and DM may be due to the low incidence of DM and small sample size.

The risk of RA, SLE, and SS was consistently increased across all of the subgroups of the PR patients stratified based on age, sex, and CCI. Of note, the risk of RA was highest in the PR patients with CCI = 0 (HR, 171.22, 95% CI, 120.47–243.34). Among the subjects with CCI = 0, the magnitudes of the relative risk for RA, SLE, or SS in the PR patients compared with the non-PR individuals were all significantly greater than those in the subjects with CCI>0 (p for interaction all <0.05). Also, the risk of RA, SLE, or SS in the PR patients was greater among those aged <65 years compared with those aged ≥65 years (p for interaction = 0.187, 0.002 and 0.008 respectively). There are some possible explanations of the interaction effects of CCI and age. First, prior studies have shown that older adults have increased production of proinflammatory cytokines and autoantibodies [26–31]. Also, low grade systemic inflammation exists in patients with diseases included in CCI, such as cancer [32], diabetes mellitus [33, 34], cardiovascular diseases [35], stroke [36], dementia [37], peripheral vascular disease [38], chronic obstructive airway diseases [39], and chronic kidney disease [40]. Given the prevailing hypothesis that unresolved low-grade systemic inflammation may drive the development of autoimmunity [41], older age (≥65 years) and comorbidities (CCI>0) may compete for the risk of autoimmune diseases associated with PR. Second, some medications, such as procainamide, hydralazine, and anticonvulsants [42, 43], may induce SLE and thus compete for the risk of SLE development associated with PR in patients with comorbidities treated with these culprit medications. Third, individuals with CCI>1 may have more ambulatory visits than those with CCI = 0, leading to an increased detection rate of another disease. Such detecting bias may also lead to a weaker association between PR and the risk of RA, SLE or SS in individuals with CCI>1.Although further studies are warranted to elucidate the mechanisms of the interaction effects of CCI and age, we may still suggest a particularly careful evaluation for the development of RA, SLE, and SS in PR patients aged less than 65 years or without comorbidities.

The strength of this study is the utilization of a nationwide population-based cohort in order to avoid a selection bias and to provide a large sample size. However, we must address some limitations. First, the accuracy of the diagnosis of PR according to the data of the claims is of concern. Although the BNHI has increased the accuracy of a diagnosis by a routine examination of the original medical records [18], the validity of the PR diagnoses needs to be tested by reviewing the medical record of selected cases in the future. Second, although the diagnosis of RA, SLE, SS, SSc, PM, and DM was validated by at least two qualified rheumatologists by checking the original medical data, the misclassification rate in the PR group may not be equal to that in the non-PR group. Such a differential misclassification bias may lead to an overestimation of the risk of autoimmune rheumatic diseases in the PR patients. Third, some potential confounding factors, including the use of tobacco and alcohol, a family history of autoimmune rheumatic diseases, BMI, and socioeconomic status were not available in the NHIRD. The lack of laboratory data, such as RF and ACPA, limited further stratified analyses according to the situation of autoantibodies. Finally, the study results may not be applied to non-Taiwanese populations.

### Conclusion

This nationwide, population-based cohort study demonstrated a strong association between PR and the development of autoimmune rheumatic diseases, including RA, SLE, SS, PM, and SSc. The relative risk of SLE and SS in PR patients compared with non-PR individuals was higher in those aged <65 years than in those aged ≥65 years. The relative risk of RA, SLE, and SS in PR patients compared with non-PR individuals was higher in those with CCI = 0 than in those with CCI >1. However, the lack of validation of PR diagnoses by reviewing original records of selected cases is a major limitation of this study. Further genetic, clinical, and immunological studies are warranted to elucidate the role of PR in the development of RA, SLE, SS, PM, and SSc and to identify the associated risk factors.

## Supporting information

S1 Data Data of the PR group and the non-PR group. (SAV)
